# Navigating the Psychosocial Journey: A Meta‐Ethnographic Review of the Psychosocial Needs and Experiences of Children and Adolescents (0–19 Years) Diagnosed With Cancer Across the Care Continuum

**DOI:** 10.1002/pon.70582

**Published:** 2026-08-18

**Authors:** Alyssa Ebert, Carolyn Ee, Oluwaseyifunmi Andi Agbejule, Michael Osborn, Murray Turner, Catherine Paterson

**Affiliations:** ^1^ Flinders University College of Health and Enablement Flinders LiveWell Cancer Research Centre Caring Futures Institute Adelaide Australia; ^2^ Central Adelaide Local Health Network Adelaide Australia; ^3^ Michael Rice Centre for Haematology and Oncology Women's and Children's Hospital North Adelaide Australia; ^4^ Youth Cancer Service SA/NT Royal Adelaide Hospital Adelaide Australia; ^5^ School of Medicine Adelaide University Adelaide Australia; ^6^ South Australian Comprehensive Cancer Network Commission on Excellence and Innovation in Health Adelaide Australia; ^7^ Faculty of Health University of Canberra Canberra Australia

**Keywords:** cancer survivorship, developmental psychology, meta‐ethnography, paediatric oncology, psychosocial needs, qualitative synthesis

## Abstract

**Background:**

Survival rates for childhood cancer have improved; however, psychosocial challenges remain across diagnosis, treatment, and survivorship. Existing qualitative studies typically examine a single phase of care, limiting understanding of how psychosocial needs persist and change over time.

**Aim:**

This meta‐ethnographic review synthesised qualitative evidence on the psychosocial needs and experiences of children and adolescents (0–19 years) diagnosed with cancer across the care continuum and examined how these needs evolve across phases.

**Method:**

A meta‐ethnographic synthesis was conducted following Noblit and Hare's approach and reported in accordance with eMERGe guidance. Searches of six databases (Scopus, MEDLINE, Web of Science, PsycINFO, CINAHL, and Google Scholar) identified 31 qualitative studies published between January 2009 and February 2025. First‐ and second‐order constructs were extracted, coded by age group and continuum phase, and integrated into a line‐of‐argument synthesis.

**Results:**

Six psychosocial domains consistently shaped children's needs and experiences: family, relationships, psychosocial care, emotions, control, and information needs. These domains remained consistent across diagnosis, treatment, and survivorship but differed in expression over time. Diagnosis was characterised by emotional shock, uncertainty, and reliance on parental containment. During treatment, children experienced procedural distress, disrupted peer relationships, and fluctuating autonomy. Survivorship involved fear of relapse, challenges with reintegration, and inconsistent psychosocial follow‐up. Evidence gaps were identified for toddlers' and school‐aged children's needs in survivorship.

**Conclusion:**

This synthesis highlights the prehabilitation phase as a potential window for early psychosocial intervention and supports the development of phase‐responsive, developmentally responsive psychosocial care pathways extending into survivorship.

## 1 Background

Advances in paediatric oncology have substantially improved survival, with approximately 80% of children now surviving 5 years after diagnosis [1]. As a result, a growing population of children and adolescents now traverse the full cancer care continuum, encompassing diagnosis, treatment, and survivorship. Despite these improvements, psychosocial morbidity remains prevalent across all phases of care. Up to one quarter of children experience clinically significant anxiety, depression, or post‐traumatic stress, particularly in the period surrounding diagnosis [2]. Long‐term survivors (5–20 years post diagnosis) also demonstrate elevated vulnerability to ongoing psychological distress, challenges with school and social reintegration, and reduced emotional resilience [3, 4, 5, 6]. Psychosocial demands vary substantially across the cancer care continuum. Diagnosis often represents an abrupt rupture from normal life, characterised by emotional shock, rapid information processing, and disruption of daily routines [7]. Treatment introduces ongoing procedural distress, altered physical appearance, separation from peers and school, and prolonged dependence on caregivers [8]. Survivorship presents a different set of challenges, including fear of relapse, identity renegotiation, academic disruption, and navigating late effects in the context of reduced clinical support [4, 9]. Although these psychosocial challenges are well documented within individual phases, it remains unclear how children's psychosocial needs persist and change as they move across the continuum.

Children's psychosocial needs are further shaped by developmental stage [10, 11, 12]. Coping capacity, communication skills, emotional regulation, and understanding of illness change markedly across childhood and adolescence, influencing how psychosocial stressors are experienced and expressed [13, 14, 15]. Infants and toddlers rely heavily on caregivers for emotional regulation and security [16, 17, 18], school‐aged children benefit from concrete explanations and reassurance [19, 20], and adolescents increasingly seek autonomy while navigating more complex emotional and social stressors [21]. Failure to account for these developmental differences risks mismatched psychosocial care and missed opportunities to support adaptive coping [12, 22, 23].

Existing reviews predominantly examine psychosocial experiences within isolated phases of care or focus on specific age groups, limiting understanding of continuity and change across the cancer care continuum [7, 24, 25]. As a result, children and families may experience gaps in psychosocial support at key transition points, including diagnosis, treatment initiation, treatment completion, and reintegration into school and community life [12, 22, 23]. These gaps include limited anticipatory guidance, inconsistent developmentally appropriate information, inadequate caregiver support, and fragmented survivorship follow‐up [12, 23]. Psychosocial needs, defined here as the emotional, social, informational, and behavioural requirements arising from the impact of cancer and its treatment on daily functioning and wellbeing [26], are multidimensional and likely to shift in both form and priority across the cancer care continuum.

**FIGURE 1:**
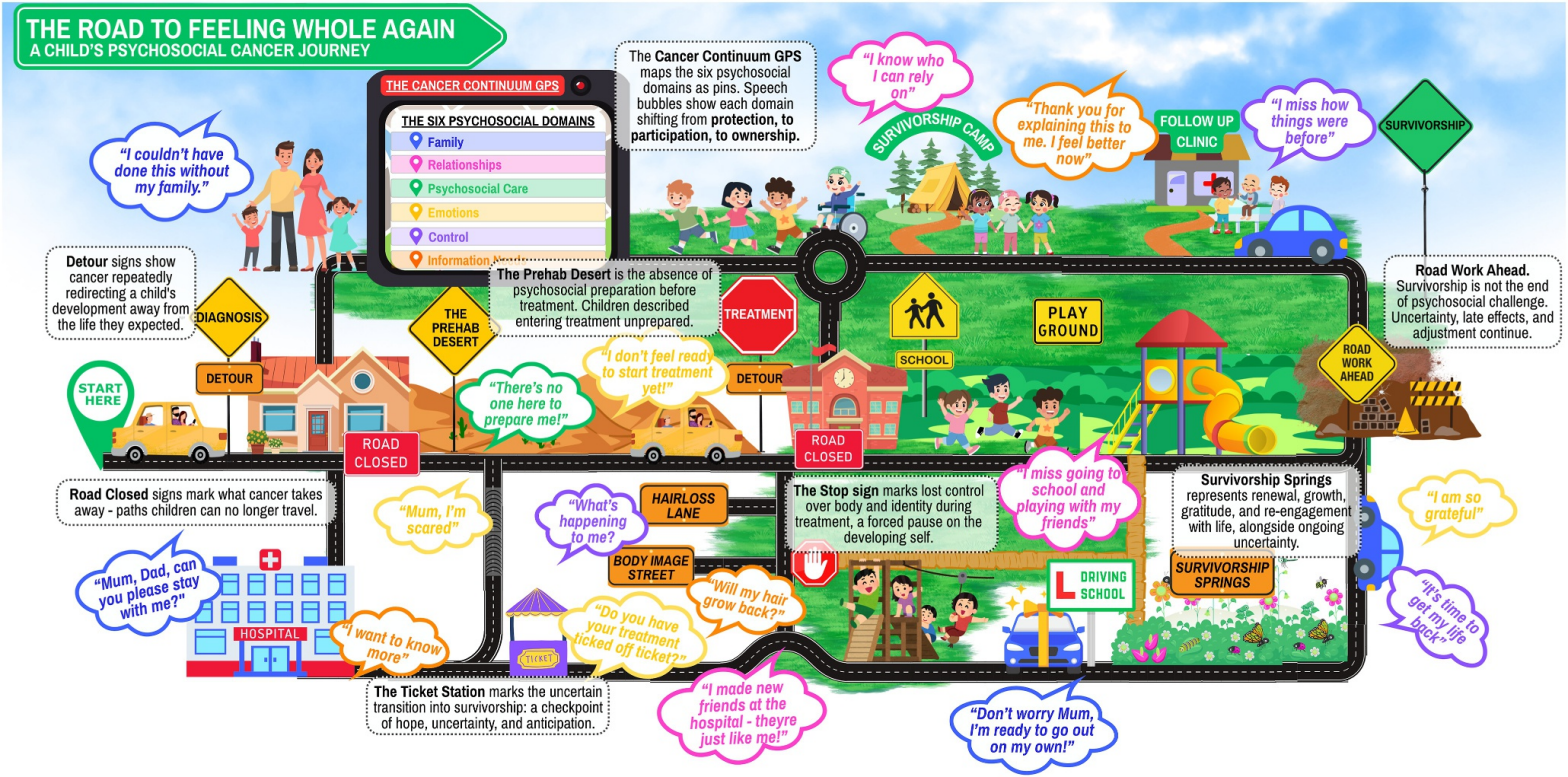
The road to feeling whole again; the cancer continuum GPS represents the six psychosocial domains identified through meta‐ethnographic synthesis (family, relationships, psychosocial care, emotions, control, and information needs) as routes within a roadmap of the cancer care continuum. The journey progresses through diagnosis, treatment, and survivorship, with landmarks symbolising recurring psychosocial experiences reported across studies. Detours and road closures represent disruptions to typical developmental trajectories; the prehab desert reflects the absence of psychosocial preparation before treatment; hair loss lane and body image street represent treatment‐related threats to identity and self‐image; the Ticket Station symbolises transition into survivorship; Road Work Ahead reflects persistent challenges after treatment completion; and Survivorship Springs represents recovery, re‐engagement, and hope. Speech bubbles are illustrative examples derived from participant accounts, capturing how psychosocial needs and voices shift across the cancer care continuum: from fear and confusion at diagnosis (‘mum, i'm scared’, ‘what's happening to me?’), to loss, isolation, and unpreparedness during treatment (‘there's no one here to prepare me’, ‘i don't feel ready to start treatment yet’, ‘i miss going to school and playing with my friends’), to growing independence and gratitude in survivorship (‘don't worry mum, i'm ready to go out on my own’, ‘i feel better now’, ‘i'm so grateful’). These examples demonstrate how psychosocial needs evolve from dependence and protection toward participation and increasing ownership across the continuum. The overall arc moves from protection → participation → ownership, visually conveyed through the road progressing from a blocked, detour‐filled path toward open, flowering terrain.

Psychosocial prehabilitation, defined as proactive emotional and coping preparation delivered at or shortly after diagnosis, has gained increasing attention in adult oncology and other chronic illness contexts but remains underexplored in paediatric oncology (Prehab Desert, Figure 1) [8, 27, 28, 29]. Although implementing psychosocial support during diagnosis is challenging due to rapid treatment initiation, early psychosocial patterns formed during this period may influence how children and families adapt during treatment and survivorship [29]. Understanding how psychosocial needs evolve across phases is therefore critical for identifying opportunities to intervene early and prevent maladaptive trajectories [26, 30].

Accordingly, this meta‐ethnographic review aimed to synthesise qualitative evidence on the psychosocial needs and experiences of children and adolescents (0–19 years) diagnosed with cancer across diagnosis, treatment, and survivorship. Specifically, the review sought to generate a line‐of‐argument synthesis explaining how psychosocial needs persist yet change in expression across the cancer care continuum, with the goal of informing developmentally responsive psychosocial care and future prehabilitation intervention design. Unlike individual primary studies, which capture psychosocial experiences within a single phase or population, meta‐ethnography enables the generation of third‐order conceptual understanding by translating and synthesising interpretations across studies [31]. The protection‐participation‐ownership model presented here is precisely this kind of contribution: a conceptual synthesis that could not emerge from any individual study but arises from reading across the full body of qualitative evidence.

Given paediatric psychologists' central role in assessment, intervention, and continuity of psychosocial care across the cancer care continuum, synthesising how children's psychosocial needs persist and change across phases has direct implications for paediatric psychology practice, service design, and intervention timing.

## 2 Methods

This meta‐ethnographic review followed Noblit and Hare's [31] seven‐phase approach, encompassing study selection and immersion, reciprocal and refutational translation, synthesis, and line‐of‐argument development. The review was further guided by Sattar et al.’s [32] stepwise recommendations to enhance transparency and methodological clarity. Reporting followed the Enhancing Meta‐Ethnography Reporting Guidelines (eMERGe; 34). See Supporting Information S1: File 1 for completed checklist. The review protocol was registered with the International Prospective Register of Systematic Reviews (PROSPERO; registration number: CRD420260626125).

Meta‐ethnography enables interpretive synthesis across qualitative studies by translating first‐order constructs (participants' accounts) and second‐order constructs (authors' interpretations) into third‐order constructs that generate new conceptual understandings [31]. This approach was well suited to examining how psychosocial needs persist yet change in expression across the cancer care continuum, as it privileges relational, contextual, and developmental meaning‐making. The synthesis was informed by an interpretivist epistemology, recognising psychosocial experiences as co‐constructed within family, clinical, and sociocultural contexts.

### 2.1 Search Strategy

The search strategy was developed in collaboration with an academic research librarian (MT) through a series of one‐on‐one meetings. Search terms were developed from scratch and informed by MeSH terms across four key concepts: paediatric cancer, psychosocial needs or experiences, cancer care continuum (diagnosis, treatment, survivorship), and qualitative methodology. An initial scoping search was conducted by MT and imported into EndNote. Searches were then conducted across six databases—Scopus, MEDLINE, Web of Science, PsycINFO, CINAHL, and Google Scholar. Google Scholar was searched via Publish or Perish as a supplementary search to capture sources not indexed in the structured databases; due to Google Scholar's limited Boolean functionality, a simplified keyword search was employed rather than the full structured string used across other databases. Searches were limited to English‐language publications involving human participants aged 0–19 years and published between January 2009 and February 2025. Date (January 2009‐ February 2025) and English‐language restrictions were applied to ensure currency and align with reviewer competencies. The full search strategy for each database is provided in Supporting Information S1: File 2.

**FIGURE 2:**
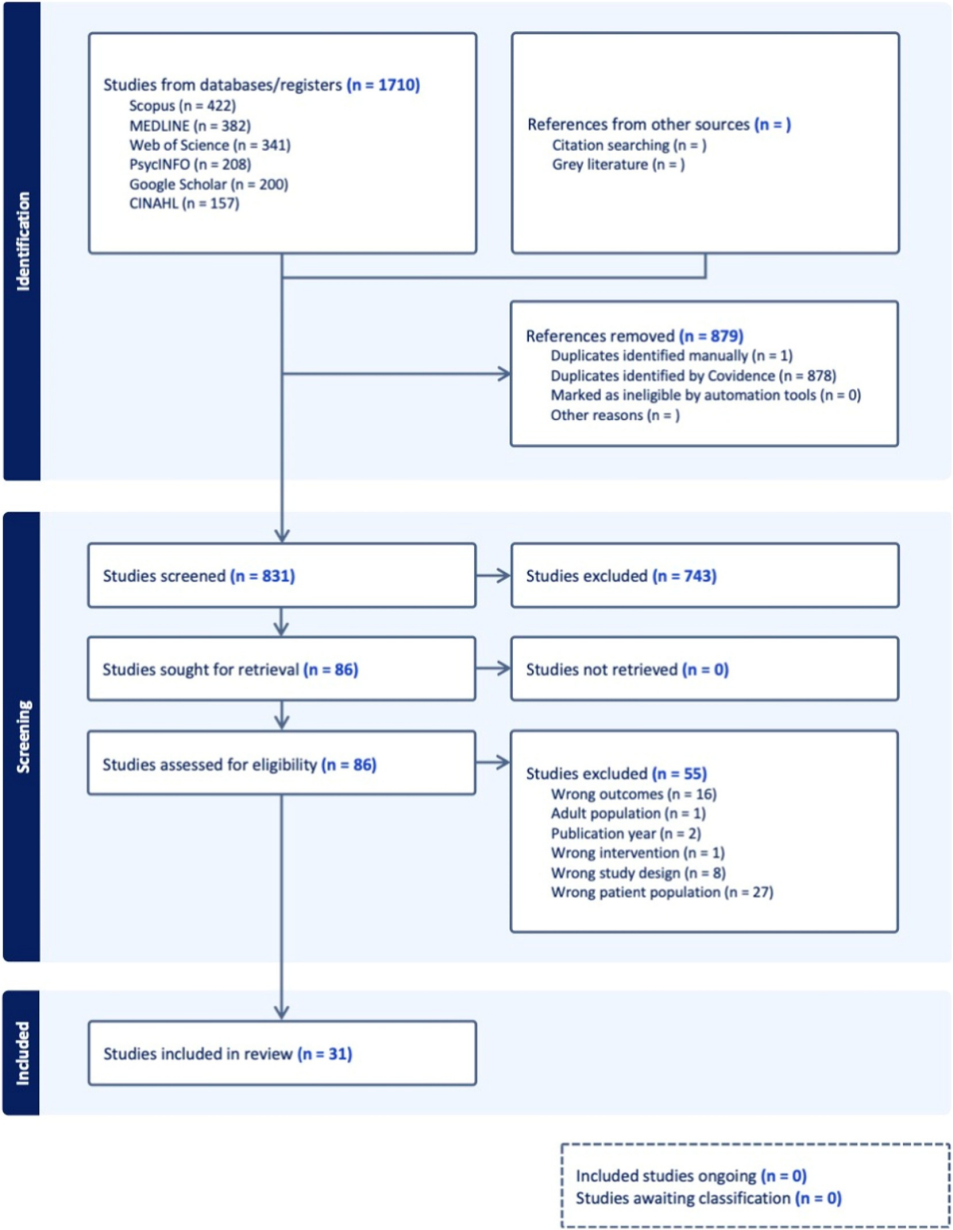
PRISMA diagram.

A total of 1710 records were imported into Covidence. Titles and abstracts were independently screened by two reviewers (AE and CP). Full‐text screening was independently conducted by two reviewers (AE and OAA). Disagreements were resolved through discussion, with a third reviewer (CP or OAA) consulted when consensus could not be reached. Thirty‐one studies met inclusion criteria (Figure 2).

### 2.2 Study Selection

Studies were eligible if they used qualitative methods and reported psychosocial experiences or supportive care needs of children and adolescents diagnosed with cancer aged 0–19 years, or their primary caregivers, during diagnosis, treatment, or survivorship. Psychosocial needs were defined as emotional, social, informational, and behavioural requirements arising from the impact of cancer and its treatment on daily functioning and wellbeing [26].

Studies were included when at least 80% of participants were aged 0–19 years to maximise relevance to paediatric experiences while avoiding unnecessary exclusion of mixed‐age samples. Quantitative studies, mixed‐methods studies without extractable qualitative findings, non‐English publications, and studies focused solely on physical outcomes were excluded. Studies addressing palliative, bereavement, or end‐of‐life care were excluded, as these contexts warrant separate synthesis due to their distinct psychosocial dynamics.

### 2.3 Quality Appraisal

Methodological quality was assessed using the Joanna Briggs Institute Critical Appraisal Checklist for Qualitative Research [33]. Two reviewers independently appraised each study (AE and CP), rating items as ‘Yes’, ‘No’, or ‘Unclear’ (Table 1). Consistent with guidance for interpretive synthesis, no studies were excluded based on quality; instead, appraisal informed interpretive caution during synthesis, with lower‐quality studies contributing to translation but weighted conservatively [61].

**TABLE 1:** Methodological quality assessment.

Qualitative study	JBI critical appraisal checklist									
	1	2	3	4	5	6	7	8	9	10
(Akbarbegloo et al. 2024) [34]	Y	Y	Y	Y	Y	N	N	Y	Y	Y
(Ang et al. 2018) [35]	Y	Y	Y	Y	Y	Y	Y	Y	Y	Y
(Arpaci et al. 2022) [36]	Y	Y	Y	Y	Y	U	U	Y	Y	Y
(Belpame et al. 2021) [12]	Y	Y	Y	Y	Y	N	N	Y	Y	Y
(Bhatt et al. 2023) [37]	Y	Y	Y	Y	Y	N	N	Y	Y	Y
(Cheng et al. 2022) [38]	Y	Y	Y	Y	Y	N	N	Y	Y	Y
(Cho et al. 2024) [39]	Y	Y	Y	Y	Y	N	N	Y	Y	Y
(Darcy et al. 2014) [40]	Y	Y	Y	Y	Y	U	U	Y	Y	Y
(Darcy et al. 2014) [41]	Y	Y	Y	Y	Y	N	N	Y	Y	Y
(Dinc et al. 2024) [42]	Y	Y	Y	Y	Y	Y	Y	Y	Y	Y
(Hildenbrand et al. 2011) [43]	Y	Y	Y	Y	Y	N	N	Y	Y	Y
(Inhestern et al. 2020) [11]	Y	Y	Y	Y	Y	Y	U	Y	Y	Y
(Jones et al. 2011) [44]	Y	Y	Y	Y	Y	N	N	Y	Y	Y
(Larsen et al. 2022) [45]	Y	Y	Y	Y	Y	N	N	Y	Y	Y
(Li et al. 2025) [46]	Y	Y	Y	Y	Y	Y	U	Y	Y	Y
(Macartney et al. 2014) [47]	Y	Y	Y	Y	Y	Y	Y	Y	Y	Y
(McKenzie et al. 2012) [48]	Y	Y	Y	Y	Y	N	N	Y	Y	Y
(Parker et al. 2023) [49]	Y	Y	Y	Y	Y	Y	N	Y	Y	Y
(Rivero‐ Vergne et al. 2011) [50]	Y	Y	Y	Y	Y	Y	N	Y	Y	U
(Seo et al. 2021) [51]	Y	Y	Y	Y	Y	N	U	Y	Y	Y
(Stinson et al. 2012) [52]	Y	Y	Y	Y	Y	N	N	Y	Y	Y
(Tenniglo et al. 2017) [53]	Y	Y	Y	Y	Y	Y	N	Y	Y	Y
(Testoni et al. 2023) [54]	Y	Y	Y	Y	Y	N	N	Y	Y	Y
(Wakefield et al. 2013) [9]	Y	Y	Y	Y	Y	N	U	Y	Y	Y
(Walker et al. 2019) [55]	Y	Y	Y	Y	Y	N	N	Y	Y	Y
(Wilford et al. 2017) [56]	U	Y	Y	Y	Y	U	N	Y	Y	Y
(Williams et al. 2015) [57]	Y	Y	Y	Y	Y	Y	U	Y	Y	Y
(Winzig et al. 2023) [58]	Y	Y	Y	Y	Y	N	N	Y	Y	U
(Wong et al. 2024) [59]	Y	Y	Y	Y	Y	N	U	Y	Y	Y
(Wu et al. 2009) [10]	Y	Y	Y	Y	Y	Y	N	Y	Y	Y
(Young et al. 2023) [60]	Y	Y	Y	Y	U	Y	Y	Y	Y	U
1	Yes
2	No
3	Unclear

*Note:* JBI Quality Assessment Checklist: 1 Is there congruity between the stated philosophical perspective and the research methodology? 2 Is there congruity between the research methodology and the research question or objectives? 3 Is there congruity between the research methodology and the methods used to collect data? 4 Is there congruity between the research methodology and the representation and analysis of data? 5 Is there congruity between the research methodology and the interpretation of results? 6 Is there a statement locating the researcher culturally or theoretically? 7 Is the influence of the researcher on the research, and vice‐versa, addressed? 8 Are participants, and their voices, adequately represented? 9 Is the research ethical according to current criteria or, for recent studies, and is there evidence of ethical approval by an appropriate body? 10 Do the conclusions drawn in the research report flow from the analysis, or interpretation, of the data?.

### 2.4 Data Extraction and Coding

Data extraction was conducted by one reviewer (AE) and cross‐checked by the research team. Extracted data included bibliographic details, study context, participant characteristics, developmental age band, cancer type, and cancer care phase. First‐order participant quotations and second‐order author interpretations were imported verbatim into the qualitative analysis software Quirkos (version 2023). Included studies reported perspectives from children, caregivers, or both. During initial coding, child‐generated data were tagged and distinguished from caregiver accounts. Where caregiver accounts described children's experiences, child‐specific data including age, diagnosis and care phase were extracted and attributed to the child where possible, to preserve the child‐centred focus of the coding framework. However, for the purposes of synthesis, all perspectives were integrated and used collectively to describe children's psychosocial experiences. For younger age groups where direct child data were unavailable, caregiver proxy reports were treated as the primary data source, with careful interpretive consideration applied during coding to acknowledge that these accounts reflect caregivers' observations and interpretations rather than children's direct voices.

Coding followed a hybrid deductive‐inductive approach informed by Fereday and Muir‐Cochrane [62] and Gale et al. [63]. An initial deductive framework of provisional domains was developed from the existing psychosocial literature prior to coding. Inductive coding then iteratively refined these domains: an initial separation of emotional experiences and positive affect was consolidated into a single emotions domain encompassing both positive and negative emotional trajectories; domains capturing longing for pre‐illness life and adjustment to ongoing change were merged into a control domain, as coding revealed these reflected a shared underlying drive to reclaim agency; an initial domain of socialisation challenges was broadened to relationships, to capture both the disruptions and the meaningful connections that emerged across studies; and information needs, initially subsumed within the control domain, was separated into a distinct domain as coding revealed it operated independently—shaping children's experiences across all phases regardless of their sense of control. The six final domains—family, relationships, psychosocial care, emotions, control, and information needs—therefore reflect both deductive grounding and inductive refinement.

Each construct was also coded by developmental age band and cancer care phase to enable systematic comparison across developmental stages and the care continuum. Four age bands were applied, reflecting well‐established theoretical periodisation in developmental psychology [10, 11, 12, 13, 14, 15]: the 0–3 year band corresponds to Piaget's sensorimotor and early preoperational stages and Erikson's trust versus mistrust stage, characterised by primary attachment formation and caregiver co‐regulation; the 4–6 year band covers the preoperational to early concrete operational transition, marked by emerging symbolic thinking and the initiative versus guilt psychosocial task; the 7–12 year band aligns with concrete operational thinking and Erikson's industry versus inferiority stage, during which mastery‐oriented coping and peer comparison become salient; and the 13–19 year band corresponds to formal operational thinking and Erikson's identity versus role confusion stage, during which autonomy‐seeking and future orientation become central developmental concerns. We defined continuum phases as: Diagnosis (diagnosis → first treatment), Active Treatment (first cancer therapy → protocol‐defined off‐therapy date or documented shift to surveillance), Survivorship (off‐therapy → 12 months+) [64].

### 2.5 Data Synthesis

According to meta‐ethnography methodology [31], concepts were compared across studies to identify relationships and preliminary conceptual clusters and descriptive labels. Early patterns suggested that psychosocial needs commonly clustered within the six domains and persisted across phases but changed in expression. Domain labels were iteratively refined throughout analysis through domain merges, relabelling, and one domain split. The final six domains reflect both deductive grounding and demonstrable inductive refinement rather than a priori imposition. Reciprocal and refutational translation identified convergent findings while also exploring any contradictions. Where contradictions emerged, such as parental buffering being described as both protective and constraining, these were examined in relation to developmental stage, illness context, and study design.

Next, third‐order interpretations were developed by integrating analytic memos across domains, developmental stages, and care phases. Emerging interpretations were iteratively checked against first‐ and second‐order data to ensure interpretive fidelity. Only constructs demonstrating conceptual coherence across studies informed the final line‐of‐argument synthesis.

To illustrate the translation process, a first‐order construct from one included study—a child describing independently applying anaesthetic cream before a procedure: *‘Put it on at home… so that it won't hurt, put on the tape myself*’—was interpreted by the study authors as *‘taking command through participation in care’* (second‐order construct). Across studies, this construct translated into a third‐order understanding of children's need for control over procedures as a mechanism for feeling capable and accountable, contributing to the control domain. This process was repeated across all 31 studies, with first‐ and second‐order constructs systematically compared and translated into one another before third‐order interpretations were developed. Analytic memos documenting interpretive decisions and domain reasoning were written throughout coding and reviewed at weekly team meetings; rigour strategies including independent coding checks and reflexive memoing are detailed in the Rigour section below.

Contradictions were retained where meaningful rather than simplified. Only constructs that demonstrated cross‐study coherence formed part of the line‐of‐argument model describing how psychosocial needs remained consistent in structure but shifted in expression across diagnosis, treatment and survivorship. Rather than summarising each study individually, the results present an interpretive synthesis integrating convergent and divergent findings across studies, developmental stages, and care phases.

### 2.6 Rigour

Rigour was enhanced through maintenance of a detailed audit trail documenting coding decisions, analytic memos, and interpretive discussions. An independent review of 20% of the coded dataset identified fewer than 5% discrepancies, all of which were resolved through discussion. Reflexivity was maintained throughout the synthesis given the clinical backgrounds of the research team. The primary reviewer (AE) is a radiation therapist with direct experience in paediatric oncology; OAA is a Research Fellow in Cancer Survivorship with a background in radiation therapy in a non‐clinical research capacity; CP is a Professor of Cancer Nursing and Nurse Practitioner with extensive expertise in cancer survivorship research; CE is an Associate Professor in Cancer Survivorship and Primary Care with a background in general and integrative medicine; and MO is a paediatric oncologist. These backgrounds brought valuable clinical insight—including familiarity with procedural distress, paediatric oncology care pathways, cancer nursing, survivorship, and primary care—but also carried the risk of privileging clinician perspectives over children's own accounts. To manage this, reflexive memoing was used by the primary reviewer (AE) throughout coding to surface and bracket clinical assumptions. Weekly team meetings, conducted over months, interpretations were actively interrogated by all reviewers, with deliberate effort to foreground first‐order participant data—returning consistently to original quotes to ensure interpretive claims remained grounded in children's and families' accounts rather than clinical expectation. OAA's non‐clinical research role provided an important counterbalance, offering a perspective less anchored in direct clinical practice and grounding interpretive discussions in the data. The diversity of disciplinary backgrounds across the team was considered a reflexive asset, enabling assumptions held by any one reviewer to be surfaced and challenged collectively.

Member checking is not an applicable rigour strategy for meta‐ethnographic synthesis, which works with published interpretations from separately conducted primary studies rather than direct participant data [31, 61]. In lieu of member checking, credibility and confirmability of the synthesis were addressed through multiple complementary strategies: an independent coding check conducted across 20% of the dataset yielded fewer than 5% discrepancies; an audit trail documented all analytic and interpretive decisions throughout the synthesis; reflexive memoing was used to surface and bracket clinical assumptions; and regular team interpretive discussions ensured that emerging third‐order constructs were interrogated and refined collectively before being accepted into the synthesis. Together, these strategies provide a robust credibility framework appropriate to meta‐ethnographic methodology.

## 3 Results

Thirty‐one qualitative studies met inclusion criteria (Table 2). The studies varied in design, sample characteristics, and clinical context, and represented perspectives from infancy through adolescence. Notable evidence gaps included limited representation of toddlers and preschool‐aged children (e.g., 2–6 years old) during treatment and a lack of evidence describing school‐aged survivors' information needs. Studies spanned the full cancer care continuum, including diagnosis (*n* = 3), treatment (*n* = 9), and survivorship (*n* = 19).

**TABLE 2:** Study characteristics.

Author (Year)	Country	Participants	Age range (years)	Cancer diagnosis	Study design	Care phase
[34]	Iran	Survivors, parents, professionals (*n* = 27)	12–19	Leukaemia; sarcoma; brain tumour; wilms tumour; retinoblastoma	Semi‐structured interviews; qualitative descriptive	Survivorship
[35]	Singapore	Adolescents with cancer (*n* = 10)	10–16	Leukaemia; brain tumour; ovarian tumour; lymphoma	Semi‐structured interviews; qualitative descriptive	Treatment
[36]	Turkey	Adolescent leukaemia survivors (*n* = 16)	12–19	Acute lymphoblastic leukaemia	Semi‐structured interviews; qualitative descriptive	Survivorship
[12]	Belgium	Childhood cancer survivors (*n* = 21)	0–12	Leukaemia; bone tumour; lymphoma; brain tumour	Semi‐structured interviews; qualitative descriptive	Survivorship
[37]	United States	Caregivers of transplant recipients (*n* = 19)	13–18	Leukaemia; myelodysplastic syndromes	Semi‐structured interviews; qualitative descriptive	Survivorship
[38]	China	Children with cancer (*n* = 8); caregivers (*n* = 24)	4–14	Leukaemia; brain tumour; sarcoma	Interviews; phenomenology	Treatment
[39]	South Korea	Survivors and parents (*n* = 19)	13–18	Acute lymphoblastic leukaemia	Semi‐structured interviews; qualitative descriptive	Survivorship
[40]	Sweden	Children with cancer and parents (*n* = 26)	1–5	Leukaemia; solid tumours	Interviews; qualitative longitudinal	Treatment, survivorship
[41]	Sweden	Children with cancer and parents (*n* = 36)	1–5	Leukaemia; wilms tumour; brain tumour	Interviews; qualitative descriptive	Diagnosis
[42]	Turkey	School‐aged children with cancer (*n* = 16)	8–13	Leukaemia; neuroblastoma; sarcoma	Semi‐structured interviews; qualitative descriptive	Diagnosis, treatment
[43]	United States	Child‐caregiver dyads (*n* = 15)	6–8	Leukaemia; brain tumour; lymphoma	Semi‐structured interviews; qualitative descriptive	Treatment
[11]	Germany	Parents of survivors (*n* = 49)	10–17	Leukaemia; CNS tumours	Semi‐structured interviews; qualitative descriptive	Survivorship
[44]	United States	Adolescent survivors (*n* = 12)	12–20	Mixed diagnoses	In‐depth interviews; qualitative descriptive	Survivorship
[45]	Denmark	Childhood cancer survivors (*n* = 22)	10–18	Leukaemia; lymphoma; CNS tumours; solid tumours	Semi‐structured interviews; qualitative descriptive	Survivorship
[46]	China	Children with cancer (*n* = 20)	5–10	Leukaemia; lymphoma; hepatoblastoma	Semi‐structured interviews; qualitative descriptive	Treatment
[47]	Australia	Brain tumour survivors (*n* = 12)	9–18	Astrocytoma; medulloblastoma	Semi‐structured interviews; interpretive descriptive	Survivorship
[48]	Australia	Parents of children with cancer (*n* = 11)	5–17	Leukaemia; sarcoma; solid tumours	Semi‐structured interviews; grounded theory	Survivorship
[49]	Australia	Survivors and family members (*n* = 55)	0–14	Leukaemia; sarcoma; neuroblastoma	Semi‐structured interviews; qualitative descriptive	Survivorship
[50]	Spain	Children with cancer and mothers (*n* = 14)	6–17	Acute lymphoblastic leukaemia	In‐depth interviews; phenomenology	Survivorship
[51]	South Korea	Children with cancer and caregivers (*n* = 52)	6–12	Haematological malignancies	Semi‐structured interviews; qualitative descriptive	Treatment
[52]	Canada	Adolescents, parents, providers (*n* = 29)	13–18	Leukaemia; brain tumour; solid tumours	Interviews and focus groups; grounded theory	Treatment, survivorship
[53]	Netherlands	Children with cancer and parents (*n* = 29)	3–18	Leukaemia; brain tumour; bone tumour	Focus groups; qualitative descriptive	Treatment
[54]	Italy	Adolescents with cancer and caregivers (*n* = 6)	11–17	Sarcoma; leukaemia; lymphoma	Narrative interviews; qualitative descriptive	Diagnosis
[9]	Australia	Survivors and families (*n* = 70)	12–18	Leukaemia; lymphoma; sarcoma; CNS tumours	Semi‐structured interviews; qualitative descriptive	Survivorship
[55]	United States	Adolescent survivors (*n* = 29)	12–18	Leukaemia; lymphoma; bone tumours	Semi‐structured interviews; qualitative descriptive	Survivorship
[56]	United States	Brain tumour survivors and parents (*n* = 19)	13–18	Astrocytoma; medulloblastoma; ependymoma	Semi‐structured interviews; qualitative descriptive	Survivorship
[57]	United Kingdom	Mothers of children with cancer (*n* = 15)	2–6	Acute lymphoblastic leukaemia	Semi‐structured interviews; grounded theory	Survivorship
[58]	Germany	Adolescent survivors (*n* = 19)	14–18	Leukaemia; lymphoma; brain tumour	Interviews; qualitative descriptive	Survivorship
[59]	Hong Kong	Survivors and parents (*n* = 17)	8–17	Leukaemia; sarcoma; brain tumour	Photovoice; qualitative descriptive	Survivorship
[10]	Taiwan	Adolescents with cancer (*n* = 10)	12–18	Leukaemia; lymphoma; osteosarcoma	In‐depth interviews; phenomenology	Diagnosis, treatment, survivorship
[60]	United States	Caregivers of children with cancer (*n* = 23)	0–18	Brain tumours	Semi‐structured interviews; qualitative descriptive	Treatment

*Note:* Care phase reflects the dominant focus of each study.

Abbreviations: ALL = acute lymphoblastic leukaemia; AML = acute myeloid leukaemia; CNS = central nervous system.

**FIGURE 3:**
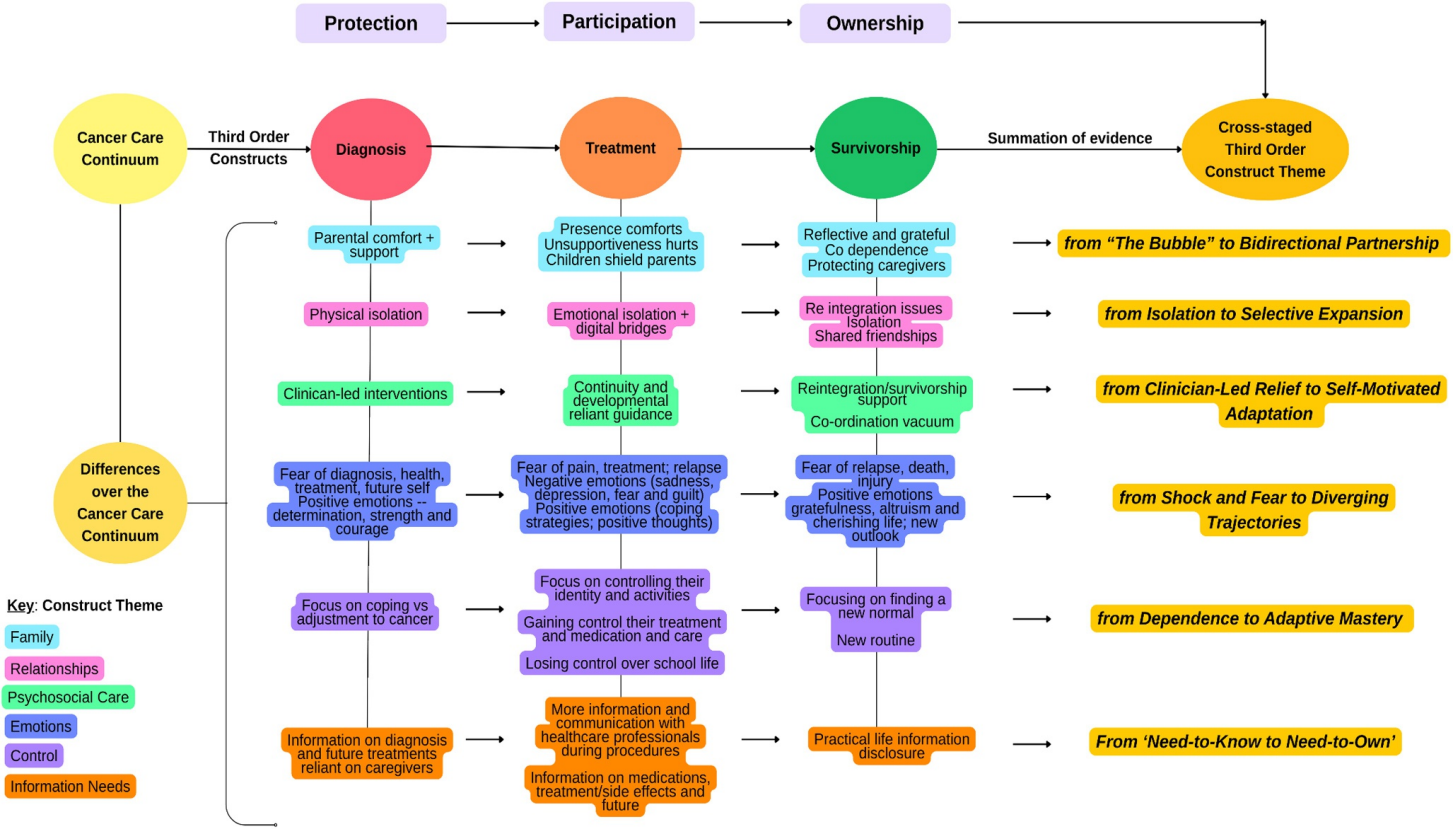
Third order construct and line of argument synthesis.

Across studies, six psychosocial domains consistently shaped children's experiences and needs: family, relationships, psychosocial care, emotions, control, and information needs. Although these domains remained consistent across diagnosis, treatment, and survivorship, their expression shifted as children progressed through the continuum. To visualise these findings, the six domains were translated into a conceptual roadmap—‘The Cancer Continuum GPS’ (Figure 1). The figure represents the psychosocial cancer journey as a developmental pathway, depicting how each domain is experienced and renegotiated across diagnosis, treatment, and survivorship. Landmarks, road signs, and character narratives were derived from recurring concepts within the included studies and illustrate the challenges, transitions, supports, and developmental tasks described by children and adolescents. Figure 3 synthesises these six trajectories into the protection‐participation‐ownership line‐of‐argument model.

Figure 3 presents the line‐of‐argument synthesis in written form, illustrating how the six domain trajectories depicted in Figure 1 were conceptually integrated to generate the protection‐participation‐ownership model. Where Figure 1 shows each domain's arc visually across the cancer care continuum, Figure 3 provides the interpretive depth behind those arcs—explaining how and why the six psychosocial domains (family, relationships, psychosocial care, emotions, control, and information needs) reconfigure in patterned ways as children move from diagnosis through treatment and into survivorship, and what that reconfiguration means at a conceptual level. The three overarching third‐order constructs, protection, participation, and ownership, are depicted as sequential yet overlapping phases, each underpinned by all six domains. Protection characterises diagnosis, during which children relied on parental containment, predictable routines, and clinician‐mediated buffering. Participation characterises treatment, during which children increasingly sought involvement in care decisions, procedural preparation, and communication. Ownership characterises survivorship, during which children worked to reclaim autonomy, manage future‐oriented uncertainty, and integrate the cancer experience into their emerging identities. The overlapping nature of the phases reflects that these shifts are developmentally contingent and non‐linear, not every child moves through each construct at the same time or in the same way. Arrows indicate the directional progression of psychosocial needs across the continuum. The figure should be read in conjunction with the Line of Argument narrative, which provides the full evidential grounding for each construct.

### 3.1 Diagnosis

Across studies, diagnosis was described as a sudden disruption to normal life, marking entry into an unfamiliar clinical world [41, 42, 54]. This phase was characterised by acute emotional shock, fear, and disruption of daily routines, alongside a strong search for stability. Family functioning centred on parental proximity and emotional containment. Younger children (0–3 years) relied heavily on physical closeness and consistent routines; Darcy et al. [41] described this as ‘embodied security’, whereby familiar sensory cues buffered clinical unfamiliarity. School‐aged children (7–12 years) similarly depended on parental reassurance, identifying parents as essential sources of courage during procedures [42]. Older children and adolescents sometimes engaged in ‘reciprocal emotional shielding’, suppressing distress to protect caregivers (speech bubbles at diagnosis, Figure 1) [42].

Diagnosis was associated with an immediate contraction of social networks. Adolescents (13–19 years) attempted to maintain pre‐diagnosis friendships but experienced significant disruption due to sudden hospitalisation and treatment demands [42]. Many described rapid relational closeness with nurses and clinicians, facilitated through humour, advocacy, and proximity [41]. Encounters with peers during hospitalisation provided shared understanding and companionship but also introduced relational grief when peers deteriorated or died [41].

Psychosocial care was most meaningful when developmentally appropriate. Younger children engaged with symbolic and sensory play‐based interventions to externalise fear, whereas adolescents employed cognitive strategies such as reframing and positive self‐talk [40, 42]. Emotional experiences across age groups were frequently described as oscillating between distress and hope [42]. Children commonly feared procedures, uncertainty about the future, and witnessing deterioration in other patients. Information needs at diagnosis were inconsistently met. Adolescents emphasised a desire for clear, direct communication addressed to them rather than filtered solely through parents [54]. Evidence describing younger children's information needs was minimal, reflecting a broader gap in the literature.

### 3.2 Treatment

During treatment, children navigated invasive procedures, prolonged hospitalisation, altered physical appearance, and sustained separation from peers and school (Hair loss Lane; Body Image Street, Figure 1). These experiences reconfigured family dynamics (care‐coordination, reduced autonomy), peer relationships (less in‐person contact, participation loss, visibility‐related stigma) and emotional regulation (greater reliance on external co‐regulation and altered sense of control). Parental presence remained emotionally stabilising across age groups. Younger children valued honest, predictable communication and expressed heightened distress when parents minimised pain or broke procedural promises [43, 51]. Some older children assumed protective emotional roles, downplaying fears to shield parents [38, 46].

Peer relationships were significantly disrupted. Digital communication enabled some maintenance of friendships; however, relational distance often increased over time [35]. Hospital peers provided shared understanding and companionship, particularly for younger children, but anticipation of returning to school was frequently associated with anxiety regarding appearance‐based stigma and social acceptance [43, 46].

Children engaged with psychosocial interventions to varying degrees. Interventions such as play therapy, journaling, distraction tools, and relaxation strategies were valued when delivered consistently by familiar clinicians [53, 60]. In contrast, rotating clinicians and brief interactions undermined continuity of support [38]. Hospitalisation was often described as confining, with some children likening the environment to imprisonment [38, 41].

Treatment also offered opportunities for developing agency through ‘micro‐autonomies’, including preparing equipment, applying numbing cream, or requesting that clinicians address them directly [38, 41]. Communication practices played a central role, with real‐time procedural narration improving predictability and reducing anticipatory anxiety [43, 46]. Information needs expanded during treatment, encompassing procedures, medications, side effects, and long‐term implications. Younger children often interpreted clinical changes idiosyncratically, whereas older children increasingly sought information about survival, late effects, and return to routine activities [51, 60].

### 3.3 Survivorship

Survivorship was characterised by a combination of relief and ongoing uncertainty. Family relationships frequently strengthened, with survivors expressing appreciation for parental and sibling support [12, 55]. Adolescents sometimes adopted reverse emotional roles, shielding parents from ongoing worry [34, 39].

Peer relationships were reshaped. Hospital‐based friendships often persisted as uniquely validating connections [36, 52] while (School; Playground, Figure 1) reintegration into school posed challenges related to academic gaps, social anxiety, and misunderstandings about late effects [56, 65]. Lingering fatigue, late effects, and parental protectiveness sometimes restricted participation in sports and social activities, complicating efforts to re‐establish normalcy [44, 66].

Psychosocial care during survivorship was inconsistent. Some survivors accessed structured follow‐up clinics offering integrated psychological and educational support [47, 49]. More commonly, families described fragmented service systems, with parents assuming responsibility for coordinating psychological care, school reintegration, and community resources [9, 37]. Treatment completion was frequently experienced as a ‘cliff edge’, marked by reduced psychosocial support (Road Work Ahead, Figure 1).

Emotional experiences during survivorship included fear of relapse, intrusive memories, identity disruption, and survivor guilt, alongside accounts of resilience, meaning‐making, and increased gratitude [52, 55]. Information needs became increasingly future‐oriented, particularly among adolescents seeking guidance regarding relapse risk, fertility, sport participation, lifestyle decisions, and long‐term health [59, 65]. The survivorship evidence base was predominantly adolescent‐focused; evidence for children aged 0–3 years was absent across all included studies, representing a critical developmental gap.

Together, these findings demonstrate that while core psychosocial domains persist across the cancer care continuum, their expression shifts in patterned, developmentally contingent ways. To integrate these patterns, a line‐of‐argument synthesis was developed to explain how children's psychosocial needs evolve from diagnosis through treatment and into survivorship.

### 3.4 Line of Argument

This meta‐ethnographic synthesis generated a line‐of‐argument explaining how children's psychosocial needs remain structurally consistent yet change in expression across diagnosis, treatment, and survivorship. Six interrelated psychosocial domains; family, relationships, psychosocial care, emotions, control, and information needs; shaped experiences across all phases. As children progressed through the cancer care continuum, these domains were reconfigured in patterned, developmentally contingent ways. Specifically, psychosocial needs shifted from protection at diagnosis, to participation during treatment, and toward ownership in survivorship (Figure 3). At diagnosis, psychosocial needs were primarily oriented toward protection. Children relied on parental containment, predictable routines, and clinician‐mediated buffering to regulate distress and uncertainty. During treatment, psychosocial needs shifted toward participation, as children sought greater involvement in care decisions, procedural preparation, and communication. In survivorship, psychosocial needs increasingly reflected ownership, characterised by efforts to reclaim autonomy, manage future‐oriented uncertainty, and integrate the cancer experience into emerging identities. These shifts were not linear or uniform; rather, they reflected dynamic negotiation between developmental capacity, illness demands, and contextual support. The following narratives describe how each domain evolved across the care continuum and collectively contributed to the overarching developmental shift from protection to participation and ownership.

#### 3.4.1 Family—From ‘The Bubble’ to Bidirectional Partnership

Across the continuum, family functioned as the primary psychosocial anchor. During diagnosis, parental presence provided emotional containment and meaning making, particularly for younger children (speech bubbles at diagnosis, Figure 1) [41, 42]. During treatment, families negotiated evolving roles as children sought greater involvement in care while remaining emotionally dependent—with parents simultaneously functioning as protectors, advocates, and procedural participants [38, 40, 51]. In survivorship, family dynamics shifted toward renegotiation of independence, with some adolescents assuming protective roles to shield parents from ongoing worry, and parents gradually withdrawing from the hypervigilant caregiving stance adopted during treatment [34, 39, 48]. These patterns underscore the family's dual role as both emotional regulator and developmental scaffold.

#### 3.4.2 Relationships—From Isolation to Selective Expansion

Relationships outside the family were shaped by illness‐related disruption and developmental stage. Diagnosis and treatment were associated with contraction of peer networks, with hospitalisation, infection precautions, and appearance‐related stigma limiting social participation [35, 42, 46]. Hospital‐based relationships provided validation and shared understanding unavailable in pre‐diagnosis friendships, with digital communication serving as a partial bridge to maintain closeness during treatment (Hospital, Figure 1) [35, 38]. However, these same hospital‐based relationships exposed children to the deterioration and death of peers, generating a form of relational grief unique to the oncology context [35, 41, 42]. Survivorship involved effortful re‐establishment of peer identity, frequently complicated by academic disruption, changed perspectives, stigma, and lingering late effects that set survivors apart from peers who had not shared the experience [9, 44, 56]. Across phases, relational continuity emerged as a critical determinant of psychosocial adjustment.

#### 3.4.3 Psychosocial Care—From Clinician‐Led Relief to Self‐Motivated Adaptation

Psychosocial care was most effective when consistent, developmentally appropriate, and embedded within routine clinical interactions rather than delivered as a discrete add‐on service [40, 53, 60]. During diagnosis and treatment, relational continuity with familiar clinicians enhanced trust, procedural engagement, and emotional disclosure—with rotating clinicians and brief interactions consistently identified as undermining the therapeutic relationship [38, 51]. Play‐based interventions, journaling, distraction tools, and peer support programmes were valued when delivered consistently and adapted to developmental stage [40, 43, 53]. In survivorship, however, psychosocial care was frequently fragmented, with families assuming responsibility for coordinating psychological support, school reintegration, and community resources with little structured guidance [9, 48, 49]. This cliff edge was most acutely felt by adolescents navigating reintegration directly, while younger survivors' transitions were more frequently mediated by caregivers [9, 36, 55, 58]. This discontinuity contributed to unmet needs at the very transition point where developmental demands were greatest.

#### 3.4.4 Emotions—From Shock and Fear to Diverging Trajectories

Children's emotional needs persisted across all phases but varied substantially in tone, trigger, and developmental expression. At diagnosis, emotional responses were characterised by undifferentiated fear, acute shock, and anticipatory dread—frequently amplified by environmental unfamiliarity and procedural uncertainty [41, 42, 46]. Paradoxically, younger children's limited cognitive understanding of illness partially shielded them from the existential fear experienced by older children and adolescents [39, 40]. During treatment, emotional experiences became more differentiated: procedure‐specific anxiety, frustration at loss of autonomy, identity disruption related to physical appearance changes, and oscillating hope and despair were commonly described [35, 38, 43]. In survivorship, emotional trajectories diverged—with some children demonstrating post‐traumatic growth, renewed gratitude, and resilience, while others experienced persistent fear of relapse, intrusive memories, and ongoing anxiety that did not resolve with treatment completion [44, 55, 66]. Across all phases, children commonly internalised distress to protect caregivers, limiting opportunities for authentic emotional expression and underscoring the need for psychosocial spaces independent of the family unit.

#### 3.4.5 Control—From Dependence to Adaptive Mastery

Experiences of control were initially severely constrained by the rapid onset of diagnosis and the demands of treatment, with children describing loss of bodily autonomy, restricted movement, altered diet, and enforced dependence on clinical and parental decision‐making [38, 41, 42]. During treatment, children actively sought micro‐level autonomy—preparing equipment, applying numbing cream, directing the timing of procedures, and requesting that clinicians address them directly rather than through parents—as mechanisms for reasserting agency within a context of structural powerlessness [38, 40, 43]. However, the same procedural involvement could heighten anticipatory anxiety when preparation was insufficient or participation imposed rather than chosen [46]. In survivorship, control was reasserted more broadly through decision‐making related to health behaviours, educational choices, disclosure of cancer history to peers, and future planning [36, 44, 55]. These shifts highlight control not as a fixed outcome but as a developmentally contingent and contextually negotiated construct that evolved in both form and scope across the cancer care continuum.

#### 3.4.6 Information Needs—From Need‐to‐Know to Need‐to‐Own

Information needs increased in complexity and scope across the continuum, shifting from immediate and procedural at diagnosis to future‐oriented and self‐directed in survivorship. At diagnosis, children required clear, honest, and age‐appropriate explanations to reduce uncertainty and build trust—with information withheld or filtered through parents consistently associated with heightened distress and loss of control [40, 41, 42]. During treatment, information needs expanded to encompass procedures, medications, side effects, and the rationale behind clinical decisions, with children as young as nine actively seeking to understand their treatment rather than passively receiving it [38, 43, 51]. In survivorship, adolescents sought increasingly future‐oriented information regarding relapse risk, fertility, sport participation, long‐term health implications, and educational planning [36, 59, 67]. Notably, evidence describing information needs among younger children in survivorship was absent across all included studies, representing a critical gap that limits the developmentally responsive design of survivorship information programmes.

Where divergent findings were encountered across studies, these were examined as substantive rather than resolved through synthesis. A clear example is the role of parental buffering, which emerged across multiple studies as simultaneously protective and constraining. Parental emotional shielding—withholding distressing information, minimising procedural pain, and maintaining positive affect—was consistently described as a source of comfort and safety for younger children [40, 41]. However, across studies involving older children and adolescents, the same buffering behaviours were associated with reduced trust, feelings of exclusion from decision‐making, and heightened distress when withheld information was subsequently encountered [36, 39, 42]. Rather than resolving this contradiction, the synthesis treated it as a developmentally meaningful tension: parental buffering appears appropriate and protective in early childhood but becomes increasingly counterproductive as children develop the cognitive capacity and autonomy‐seeking orientation of middle childhood and adolescence. This interpretive move—treating contradiction as data rather than noise—is consistent with the refutational synthesis approach of meta‐ethnography and strengthens rather than undermines the credibility of the line‐of‐argument.

Figure 3 illustrates the integrated line‐of‐argument model depicting the developmental shift from protection to participation and ownership across the cancer care continuum.

## 4 Discussion

This meta‐ethnographic review synthesised qualitative evidence on the psychosocial needs and experiences of children and adolescents diagnosed with cancer across the cancer care continuum. Rather than conceptualising psychosocial needs as phase‐specific or static, the findings demonstrate a developmental and temporal progression in how children experience, interpret, and manage cancer‐related challenges. The six domains reflect what the data revealed ‐iteratively refined through merges, relabelling, and one split during analysis (see Methods)—rather than a priori imposition. The protection‐participation‐ownership model maps directly onto Bronfenbrenner's ecological framework [13] and Erikson's psychosocial theory of development [14]. Within Bronfenbrenner's model, the protection phase reflects children's dependence on the microsystem—particularly parental and clinician relationships—to regulate distress and maintain a sense of safety when the immediate environment becomes threatening at diagnosis. The participation phase aligns with the concept of proximal processes: the sustained, bidirectional interactions between a child and their environment through which developmental competence is built. During treatment, children's efforts to engage in procedural preparation, shared decision‐making, and communication with clinicians represent exactly this kind of active engagement with the surrounding context. The ownership phase reflects the expansion of ecological agency into broader systems, including peer networks, school, and community, as survivors seek to reclaim identity and autonomy beyond the clinical environment (Driving School; Survivorship Camp, Figure 1). Erikson's developmental stages provide a complementary lens: the industry versus inferiority stage (middle childhood) explains school‐aged children's orientation toward mastery and procedural participation during treatment, while the identity versus role confusion stage (adolescence) accounts for older children's survivorship preoccupations with self‐concept, future planning, and reintegration [14]. Together, these frameworks demonstrate that the protection‐participation‐ownership progression is not an artefact of the data, but reflects theoretically coherent shifts in children's developmental capacities and ecological positioning across the cancer care continuum.

Developmental stage emerged as a critical moderator of psychosocial needs across the continuum. Younger children relied heavily on caregiver co‐regulation and concrete communication, whereas adolescents increasingly sought autonomy, privacy, and future‐oriented information although evidence for younger age groups was drawn from a limited number of studies and these patterns should be interpreted accordingly. Where evidence permitted, failure to account for developmental differences risks underestimating distress in younger children and overlooking emerging autonomy needs in adolescents. The reliance on caregiver proxy data for younger age groups warrants particular attention. Caregiver accounts provide valuable contextual insight but may systematically differ from children's own perspectives—particularly regarding emotional experiences, procedural distress, and information needs. Findings pertaining to younger children should therefore be interpreted as reflecting caregiver‐mediated rather than directly child‐generated understanding, which has implications for how developmentally responsive care is designed and evaluated.

This synthesis also highlights diagnosis as a potential window for early psychosocial intervention, although this inference is drawn from a limited evidence base of three studies and should be interpreted with caution. While the feasibility of comprehensive psychosocial support within the prehabilitation phase may be constrained by rapid treatment initiation and emotional overload, early relational patterns, communication practices, and coping strategies established during this phase may influence psychosocial adjustment during treatment and survivorship. Psychosocial prehabilitation approaches, focused on emotional preparation, caregiver support, and anticipatory guidance, represent a theoretically warranted but empirically untested direction for future investigation—prehabilitation has not been meaningfully explored in paediatric oncology despite its establishment in adult contexts [27, 28]. The present synthesis offers a conceptual foundation from which such hypotheses may be developed and tested, but does not constitute evidence for their effectiveness or feasibility. The findings also have implications for service systems. Families frequently described fragmented psychosocial provision, limited mental health access and challenges coordinating school reintegration [9, 37, 65]. As psychosocial needs remain continuous, even when their expression changes, service models must mirror this continuity. Approaches such as psychosocial health care teams present from diagnosis to beyond survivorship, structured survivorship pathways, and integrated school liaison programs may prevent the discontinuities described by families.

Strengths include rigorous application of meta‐ethnographic methods, adherence to eMERGe reporting guidance, and diverse qualitative perspectives across the full care continuum. However, several limitations should be acknowledged. The synthesis is heavily weighted toward survivorship (*n* = 19 studies), with considerably fewer studies examining treatment (*n* = 9) and diagnosis (*n* = 3). This imbalance may have shaped the findings in important ways—the dominance of survivorship data means the six psychosocial domains and their expression are most robustly characterised for this phase, while the diagnosis and treatment phases are necessarily less fully represented. It is possible that psychosocial constructs specific to diagnosis or early treatment were underrepresented or absorbed into broader domains due to insufficient data to establish their distinctiveness. Conclusions drawn about the diagnosis phase and younger children should therefore be interpreted with caution, as the evidence base is insufficient to support strong claims about psychosocial needs during these phases. Evidence for younger children, particularly during treatment and survivorship, was also sparse, reflecting both methodological challenges and gaps in the literature. The integration of child and caregiver perspectives across included studies means the synthesis cannot be considered entirely child centred. While child‐generated data were prioritised during initial coding where distinguishable, caregiver proxy reports formed a substantial portion of the evidence base, particularly for younger children who were unable to self‐report. Caregiver accounts may not fully capture children's subjective experiences, and the degree to which findings reflect children's own perspectives versus adult interpretations varies across included studies and is inherently a limitation of the current literature. Additionally, the synthesis relied on published interpretations, which may reflect authors' analytic emphases rather than participants' experiences alone.

The protection‐participation‐ownership model is also a conceptual synthesis derived from published qualitative accounts rather than an empirically tested framework. While theoretically coherent and grounded in the included studies, its structure, sequence, and developmental contingency require prospective empirical validation, ideally through longitudinal qualitative work following children across phases, and through co‐design and testing with children, survivors, families, and clinicians, before it is used to guide service design or intervention development. Additionally, the geographic distribution of included studies also warrants comment. Only one included study was conducted in the United Kingdom, despite a substantial UK evidence base in paediatric and adolescent oncology. Our search strategy was anchored on explicit psychosocial‐domain terminology, which prioritised specificity over sensitivity and may therefore have limited retrieval of relevant qualitative work indexed under other terminology. Findings should accordingly be interpreted as potentially under‐representing UK‐based evidence, and future syntheses in this field would benefit from supplementary retrieval strategies alongside structured database searching.

Future research should prioritise longitudinal qualitative studies that follow children across multiple phases of care, with particular attention to underrepresented age groups and transitional periods and should seek methods that collect data directly from children across all age groups. Co‐designed interventions targeting psychosocial preparation at diagnosis and continuity of care into survivorship warrant further exploration.

### 4.1 Implications for Clinical Practice

This synthesis identifies three phase‐specific clinical priorities for developmentally responsive psychosocial care in paediatric oncology. These priorities are phase‐emphasised rather than phase‐bounded. Consistent with the protection‐participation‐ownership progression, in which ownership in survivorship is made possible by participation established during treatment, several needs presented here under survivorship require anticipation and active preparation from diagnosis onward. School and social reintegration should be treated as a continuous thread rather than a post‐treatment task. Information regarding fertility, late effects, and long‐term health is sought by children and adolescents during active treatment and, in the case of fertility preservation, must necessarily precede it. Preparation for the end‐of‐treatment transition should be delivered before treatment concludes, given that families in this synthesis characterised treatment completion as an abrupt cliff edge. Support for families in the graduated renegotiation of protectiveness and autonomy should likewise begin during treatment, since the hypervigilant caregiving stance adopted during active treatment was described as difficult to relinquish once it was no longer required.

### 4.2 Diagnosis: Establish Relational Safety and Caregiver Scaffolding

Psychosocial support at diagnosis should prioritise emotional containment, honest age‐appropriate communication, and structured caregiver guidance—delivered by consistent clinicians—to reduce acute distress and establish the relational foundations that buffer subsequent treatment demands. For younger children, sensory and environmental familiarisation should be incorporated into routine clinical care. Although based on limited evidence, the synthesis tentatively identifies diagnosis as a potential window for psychosocial prehabilitation, and further investigation of early intervention approaches in this phase is warranted. Early engagement with the child's school and social network should also begin at this point, establishing communication with educational staff and maintaining peer connection so that reintegration is a continuous thread rather than a post‐treatment task.

### 4.3 Treatment: Facilitate Meaningful Participation and Procedural Agency

During treatment, clinicians should actively create opportunities for children to participate in procedural preparation and care decisions in developmentally appropriate ways—including preparing equipment, directing timing, and requesting direct communication from clinicians. Psychosocial interventions should be delivered consistently by familiar staff, as rotating personnel was consistently identified as undermining therapeutic engagement and trust. School and peer connection should be actively sustained throughout treatment, through school liaison, flexible educational provision, and support for digital and in‐person contact with friends, since children in this synthesis described anticipatory anxiety about appearance‐based stigma and social acceptance well before treatment completion.

### 4.4 Survivorship: Address the Cliff Edge, Sustain and Consolidate Psychosocial Support Through Transition

Psychosocial care should not conclude at treatment completion, nor should reintegration support begin there. Structured post‐treatment support addressing fear of relapse, identity reconstruction, and the consolidation of school and social reintegration begun at diagnosis should be available to all survivors, with urgency for adolescents navigating the cliff edge transition (Follow Up Clinic, Figure 1). Integrated survivorship care models that embed longitudinal psychosocial assessment into routine follow‐up are recommended to prevent the fragmentation of support identified across included studies.

## 5 Conclusion

Children's psychosocial needs persist across diagnosis, treatment, and survivorship but change in expression in developmentally patterned ways. The protection‐participation‐ownership model generated by this synthesis offers a theoretically grounded conceptual framework for understanding these shifts and for designing continuous, developmentally responsive psychosocial care in paediatric oncology, pending empirical validation of the model in prospective research.

## Author Contributions

**Alyssa Ebert:** conceptualisation, methodology, formal analysis, investigation, data curation, writing – original draft. **Carolyn Ee:** methodology, supervision, writing – review and editing. **Oluwaseyifunmi Andi Agbejule:** methodology, supervision, writing – review and editing. **Michael Osborn:** clinical expertise, writing – review and editing. **Murray Turner:** Database search strategy and extraction. **Catherine Paterson:** conceptualisation, supervision, methodology, writing – review and editing.

## Funding

The authors have nothing to report.

## Ethics Statement

The authors have nothing to report.

## Conflicts of Interest

The authors declare no conflicts of interest.

## Supporting information


Supporting Information S1

## Data Availability

No new primary data were generated; all data analysed in this study were obtained from previously published studies and are cited within the article.
