# Supplementary material for: Navigating the Psychosocial Journey: A Meta‐Ethnographic Review of the Psychosocial Needs and Experiences of Children and Adolescents (0–19 Years) Diagnosed With Cancer Across the Care Continuum
Source: Psychooncology. 2026 Aug 18;35(8):e70582. doi: 10.1002/pon.70582 (PMC13484188; doi:10.1002/pon.70582)
Supplement: Supplementary file 1 [file PON-35-e70582-s001.docx]

## **Supplementary Files**

## **Supplementary File 1**

## *eMERGE Checklist*

## **Phase 1: Getting Started**

| Criteria | Description | Y/N |
| --- | --- | --- |
| 1. Rationale | State the rationale for conducting a meta-ethnography. | Y |
| 2. Aims & Review Questions | Clearly state the aim(s) and review question(s). | Y |

## **Phase 2: Deciding What Is Relevant**

| Criteria | Description | Y/N | |
| --- | --- | --- | --- |
| 3. Search Strategy | Describe databases, search terms, dates, and inclusion/exclusion criteria. | | Y |
| 4. Study Selection | Explain how studies were screened and assessed for relevance. | | Y |
| 5. Study Flow | Provide flow diagram or narrative of study selection. | | Y |

## **Phase 3: Reading the Studies**

| Criteria | Description | Y/N |
| --- | --- | --- |
| 6. Data Familiarisation | Describe repeated reading and identification of key concepts. | Y |

## **Phase 4: Determining How the Studies Are Related**

| Criteria | Description | Y/N |
| --- | --- | --- |
| 7. Identifying Relationships | Explain how key concepts across studies were compared. | Y |

## **Phase 5: Translating the Studies Into One Another**

| Criteria | Description | Y/N |
| --- | --- | --- |
| 8. Translation Process | Describe how concepts from one study were compared with others. | Y |
| 9. Handling Similarities & Differences | Explain how convergence/divergence was addressed. | Y |
| 10. Translation Examples | Provide illustrative examples of translations. | Y |

## **Phase 6: Synthesising Translations**

| Criteria | Description | Y/N |
| --- | --- | --- |
| 11. Synthesis Method | Explain how translations were synthesised. | Y |
| 12. Type of Synthesis | Describe whether synthesis was reciprocal, refutational, or line-of-argument. | Y |
| 13. Final Synthesis | Present the final interpretation with supporting evidence. | Y |

## **Phase 7: Expressing the Synthesis**

| Criteria | Description | Y/N |
| --- | --- | --- |
| 14. Reporting the Synthesis | Describe how the synthesis was presented and connected to existing literature. | Y |

## **Reflexivity Across Phases**

| Criteria | Description | Y/N |
| --- | --- | --- |
| 15. Researcher Reflexivity | Specify researcher backgrounds and influence. | Y |
| 16. Documentation | Describe how methodological decisions were tracked and justified. | Y |
| 17. Challenges | Explain challenges encountered and how they were resolved. |  |

## **Technical & Reporting Considerations**

| Criteria | Description | Y/N |
| --- | --- | --- |
| 18. Quality Appraisal | Report quality assessment processes (if conducted). | Y |
| 19. Reporting Transparency | Ensure reporting allows replication and clear understanding. | Y |

## **Supplementary File 2**

## *Full Search Strategy and record of database searches*

The following databases were searched on 28 January 2025: APA PsycINFO, CINAHL, Google Scholar, Medline, Scopus, and Web of Science Core collection. Searches returned a total 1,710 results. Full record of database searches below.

APA PsycINFO (via EBSCOhost)

| Search # | Concept | Search Terms | Results |
| --- | --- | --- | --- |
| #1 | Paediatric Cancer Population | ((cancer OR neoplasm* OR oncolog* OR sarcoma OR tumo#r) N5 (adolescent* OR child* OR p#ediatric OR teen* OR young OR youth OR 0-19)) | 10,581 |
| #2 | Psychosocial Needs | (((need* OR support OR wellbeing) N5 (anxiety OR cognitive OR "daily living" OR death OR depression OR developmental OR distress OR emotional OR families OR fatigue OR fear OR family OR health OR information OR “mental health” OR mortality OR pain OR psych* OR “quality of life” OR social)) OR “patient-cent#red care” OR “patient cent#red care” OR “quality of life” OR spiritual OR stigma) | 532,488 |
| #3 | Cancer Care Continuum / Changes Over Time | ((“care stages” OR “changing need*” OR “continuum of care” OR “disease trajectory” OR “evolving need*” OR “longitudinal stud*” OR “phases of treatment” OR “recovery phase*” OR “recovery stage*” OR “stage of care” OR “stages of cancer care” OR “time-based change*” OR “treatment phase*”) OR survivorship) | 125,512 |
| #4 | Qualitative studies | (((("semi-structured" OR semistructured OR unstructured OR informal OR "in-depth" OR indepth OR "face-to-face" OR structured OR guide) N3 (interview* OR discussion* OR questionnaire*)) OR “case stud*” OR ethnograph* OR fieldwork OR "field work" OR “focus group*” OR “grounded theory” OR observation* OR phenomenology OR qualitative)) | 736,150 |
| #5 |  | #1 AND #2 AND #3 AND #4 | 208 |
|  |  | Limiter: English language | 208 |

CINAHL (via EBSCOhost)

| Search # | Concept | Search Terms | Results |
| --- | --- | --- | --- |
| #1 | Paediatric Cancer Population | ((cancer OR neoplasm* OR oncolog* OR sarcoma OR tumo#r) N5 (adolescent* OR child* OR p#ediatric OR teen* OR young OR youth OR 0-19)) | 31,996 |
| #2 | Psychosocial Needs | (((need* OR support OR wellbeing) N5 (anxiety OR cognitive OR "daily living" OR death OR depression OR developmental OR distress OR emotional OR families OR fatigue OR fear OR family OR health OR information OR “mental health” OR mortality OR pain OR psych* OR “quality of life” OR social)) OR “patient-cent#red care” OR “patient cent#red care” OR “quality of life” OR spiritual OR stigma) | 645,441 |
| #3 | Cancer Care Continuum / Changes Over Time | ((“care stages” OR “changing need*” OR “continuum of care” OR “disease trajectory” OR “evolving need*” OR “longitudinal stud*” OR “phases of treatment” OR “recovery phase*” OR “recovery stage*” OR “stage of care” OR “stages of cancer care” OR “time-based change*” OR “treatment phase*”) OR survivorship) | 79,418 |
| #4 | Qualitative studies | (((("semi-structured" OR semistructured OR unstructured OR informal OR "in-depth" OR indepth OR "face-to-face" OR structured OR guide) N3 (interview* OR discussion* OR questionnaire*)) OR “case stud*” OR ethnograph* OR fieldwork OR "field work" OR “focus group*” OR “grounded theory” OR observation* OR phenomenology OR qualitative OR (MH "Qualitative Studies+")) | 649,795 |
| #5 |  | #1 AND #2 AND #3 AND #4 | 159 |
|  |  | Limiter: English language | 157 |

Google Scholar (via Publish or Perish 8)

| Search # | Concept | Search Terms | Results |
| --- | --- | --- | --- |
| #1 | Paediatric cancer patients’ psychosocial needs across the care continuum | paediatric \| cancer \| psychosocial \| needs \|qualitative | 200 |

Medline (via EBSCOhost)

| Search # | Concept | Search Terms | Results |
| --- | --- | --- | --- |
| #1 | Paediatric Cancer Population | ((cancer OR neoplasm* OR oncolog* OR sarcoma OR tumo#r) N5 (adolescent* OR child* OR p#ediatric OR teen* OR young OR youth OR 0-19)) | 161,716 |
| #2 | Psychosocial Needs | (((need* OR support OR wellbeing) N5 (anxiety OR cognitive OR "daily living" OR death OR depression OR developmental OR distress OR emotional OR families OR fatigue OR fear OR family OR health OR information OR “mental health” OR mortality OR pain OR psych* OR “quality of life” OR social)) OR “patient-cent#red care” OR “patient cent#red care” OR “quality of life” OR spiritual OR stigma) | 1,121,169 |
| #3 | Cancer Care Continuum / Changes Over Time | ((“care stages” OR “changing need*” OR “continuum of care” OR “disease trajectory” OR “evolving need*” OR “longitudinal stud*” OR “phases of treatment” OR “recovery phase*” OR “recovery stage*” OR “stage of care” OR “stages of cancer care” OR “time-based change*” OR “treatment phase*”) OR survivorship) | 298,611 |
| #4 | Qualitative studies | (((("semi-structured" OR semistructured OR unstructured OR informal OR "in-depth" OR indepth OR "face-to-face" OR structured OR guide) N3 (interview* OR discussion* OR questionnaire*)) OR “case stud*” OR ethnograph* OR fieldwork OR "field work" OR “focus group*” OR “grounded theory” OR observation* OR phenomenology OR qualitative) OR (MH "Qualitative Studies+")) | 1,815,797 |
| #5 |  | #1 AND #2 AND #3 AND #4 | 382 |
|  |  | Limiter: English language | 382 |

Scopus

| Search # | Concept | Search Terms | Results |
| --- | --- | --- | --- |
| #1 | Paediatric Cancer Population | ((cancer OR neoplasm* OR oncolog* OR sarcoma OR tumo*r) W/5 (adolescent* OR child* OR p*ediatric OR teen* OR young OR youth OR 0-19)) | 135,603 |
| #2 | Psychosocial Needs | (((need* OR support OR wellbeing) W/5 (anxiety OR cognitive OR "daily living" OR death OR depression OR developmental OR distress OR emotional OR families OR fatigue OR fear OR family OR health OR information OR “mental health” OR mortality OR pain OR psych* OR “quality of life” OR social)) OR “patient-cent*red care” OR “patient cent*red care” OR “quality of life” OR spiritual OR stigma) | 1,931,311 |
| #3 | Cancer Care Continuum / Changes Over Time | ((“care stages” OR “changing need*” OR “continuum of care” OR “disease trajectory” OR “evolving need*” OR “longitudinal stud*” OR “phases of treatment” OR “recovery phase*” OR “recovery stage*” OR “stage of care” OR “stages of cancer care” OR “time-based change*” OR “treatment phase*”) OR survivorship) | 391,989 |
| #4 | Qualitative studies | (((("semi-structured" OR semistructured OR unstructured OR informal OR "in-depth" OR indepth OR "face-to-face" OR structured OR guide) W/3 (interview* OR discussion* OR questionnaire*)) OR “case stud*” OR ethnograph* OR fieldwork OR "field work" OR “focus group*” OR “grounded theory” OR observation* OR phenomenology OR qualitative)) | 5,790,878 |
| #5 |  | #1 AND #2 AND #3 AND #4 | 428 |
|  |  | Limiter: English language | 422 |

Web of Science Core Collection

| Search # | Concept | Search Terms | Results |
| --- | --- | --- | --- |
| #1 | Paediatric Cancer Population | ((cancer OR neoplasm* OR oncolog* OR sarcoma OR tumo$r) NEAR/5 (adolescent* OR child* OR p$ediatric OR teen* OR young OR youth OR 0-19)) | 127,782 |
| #2 | Psychosocial Needs | (((need* OR support OR wellbeing) NEAR/5 (anxiety OR cognitive OR "daily living" OR death OR depression OR developmental OR distress OR emotional OR families OR fatigue OR fear OR family OR health OR information OR “mental health” OR mortality OR pain OR psych* OR “quality of life” OR social)) OR “patient-cent$red care” OR “patient cent$red care” OR “quality of life” OR spiritual OR stigma) | 1,361,610 |
| #3 | Cancer Care Continuum / Changes Over Time | ((“care stages” OR “changing need*” OR “continuum of care” OR “disease trajectory” OR “evolving need*” OR “longitudinal stud*” OR “phases of treatment” OR “recovery phase*” OR “recovery stage*” OR “stage of care” OR “stages of cancer care” OR “time-based change*” OR “treatment phase*”) OR survivorship) | 199,681 |
| #4 | Qualitative studies | (((("semi-structured" OR semistructured OR unstructured OR informal OR "in-depth" OR indepth OR "face-to-face" OR structured OR guide) NEAR/3 (interview* OR discussion* OR questionnaire*)) OR “case stud*” OR ethnograph* OR fieldwork OR "field work" OR “focus group*” OR “grounded theory” OR observation* OR phenomenology OR qualitative)) | 3,991,925 |
| #5 |  | #1 AND #2 AND #3 AND #4 | 342 |
|  |  | Limiter: English language | 341 |
